# Effects of Beverages on Alcohol Metabolism: Potential Health Benefits and Harmful Impacts

**DOI:** 10.3390/ijms17030354

**Published:** 2016-03-09

**Authors:** Fang Wang, Yu-Jie Zhang, Yue Zhou, Ya Li, Tong Zhou, Jie Zheng, Jiao-Jiao Zhang, Sha Li, Dong-Ping Xu, Hua-Bin Li

**Affiliations:** 1 Guangdong Provincial Key Laboratory of Food, Nutrition and Health, School of Public Health, Sun Yat-Sen University, Guangzhou 510080, China; missingfeng@yeah.net (F.W.); zhyujie3@mail2.sysu.edu.cn (Y.-J.Z.); zhouyue3@mail2.sysu.edu.cn (Y.Z.); saferide@126.com (Y.L.); zwky740359@163.com (T.Z.); zhengj37@mail2.sysu.edu.cn (J.Z.); zhangjj46@mail2.sysu.edu.cn (J.-J.Z.); xudpsysu@126.com (D.-P.X.); 2 School of Chinese Medicine, The University of Hong Kong, Hong Kong 999077, China; lishasl0308@163.com; 3 South China Sea Bioresource Exploitation and Utilization Collaborative Innovation Center, Sun Yat-Sen University, Guangzhou 510006, China

**Keywords:** nonalcoholic beverages, alcohol metabolism, hepatoprotection, harmful impact

## Abstract

Nonalcoholic beverages are usually consumed accompanying alcoholic drinks, and their effects on alcohol metabolism are unclear *in vivo*. In this study, the effects of 20 nonalcoholic beverages on alcohol metabolism and liver injury caused by alcohol were evaluated in mice. Kunming mice were orally fed with alcohol (52%, *v*/*v*) and beverages. The concentrations of ethanol and acetaldehyde in blood as well as the activities of alcohol dehydrogenase (ADH) and aldehyde dehydrogenase (ALDH) in liver were assessed to indicate alcohol metabolism. The levels of aspartate aminotransferase (AST) and alanine transaminase (ALT) in serum as well as the levels of malonaldehyde (MDA) and superoxide dismutase (SOD) in liver were measured to reflect the alcohol-induced liver injury. The results showed that the treatment of soda water, green tea and honey chrysanthemum tea could accelerate ethanol metabolism and prevent liver injuries caused by alcohol when companied with excessive alcohol drinking. They might be potential dietary supplements for the alleviation of harmful effects from excessive alcohol consumption. On the contrary, some beverages such as fresh orange juice and red bull are not advised to drink when companied with alcohol consumption due to their adverse effects on ethanol induced liver injury.

## 1. Introduction

Consumption of alcohol is rapidly increasing in the world. Epidemiological studies have shown low-to-moderate alcohol consumption is beneficial for several cardiovascular diseases and other diseases such as diabetes mellitus and pancreatitis [1,2,3]. However, excessive drinking does much harm to human health, causing hangover symptoms, alcoholic hepatitis, cirrhosis, and hepatocarcinoma [4,5,6]. In addition, alcohol use disorder caused by long term alcohol drinking is a detrimental disease that affects a large population [7]. Furthermore, the yearly health care and economic costs associated with alcohol are staggering and considerable.

Alcohol is eliminated through its metabolic degradation via multiple enzymatic pathways and non-enzymatic pathways. More than 90% of alcohol is metabolized in the liver, with small fractions being excreted in the breath (0.7%), sweat (0.1%), and urine (0.3%) [8]. Ethanol is first metabolized into acetaldehyde mainly through alcohol dehydrogenase (ADH), then acetaldehyde is converted into acetic acid with catalysis of mitochondrial aldehyde dehydrogenase (ALDH) [8,9]. The primary mediators of adverse effects caused by excessive alcohol consumption appear to be oxidative stress and ethanol’s first metabolite, acetaldehyde [10]. Acetaldehyde is responsible for unpleasant effects such as nausea, vomiting, tachycardia, and hypotension [11]. The studies have confirmed that several factors may affect the metabolism of ethanol, such as food and drink consumption, genetic polymorphism of ethanol-metabolizing enzymes, or medications which interfere with the activity of the metabolizing enzymes and/or with the absorption of ethanol [12,13]. The consumption of alcoholic beverages is often accompanied by some food and drinks such as meats, vegetables, fruits, and non-alcoholic beverages. Li *et al.* [14] have evaluated the effects of 57 kinds of non-alcohol beverages including herbal infusions, tea, and carbonated beverages on ADH and ALDH activities *in vitro*. The results showed that several beverages could markedly increase/reduce ADH and ALDH activities. It is therefore speculated that these beverages might have effects on the ethanol metabolism *in vivo*. Thus, it is worth attempting to investigate the effects of different non-alcoholic beverages on alcohol metabolism *in vivo*, including potential health benefits and harmful impacts. It is full of significance to look for some effective beverages capable of reducing the toxicity of alcohol and other beverages which are inappropriate to drink after alcohol consumption.

In this study, 20 non-alcoholic beverages, including herb infusions, tea, and carbonated beverages, which are consumed frequently in China, were selected. Their effects on alcohol metabolism and potential health benefits and harmful impacts when accompanied with alcohol consumption were systematically evaluated using mice as test models. This work could supply new information on the effects of non-alcoholic beverages on alcohol metabolism and human health for nutritionists and the general public to reduce the harm from excessive alcohol consumption.

## 2. Results and Discussions

### 2.1. Effects of Beverages on Concentrations of Ethanol and Acetaldehyde in Blood

The levels of blood alcohol and acetaldehyde are rapidly elevated 2 h after alcohol consumption when compared with the normal group (untreated group) (Table 1). The blood ethanol levels ranged between 1300 and 2200 mg/L. The majority of these beverages did not markedly affect the content of ethanol in blood except that one beverage called honey citron tea (1332.72 ± 249.50 mg/L, *p* < 0.05) could significantly decrease the concentration in blood. The levels of blood acetaldehyde ranged between 58 and 87 mg/L. Honey citron tea (84.88 ± 16.75 mg/L, *p* < 0.05), red bull (76.12 ± 2.60 mg/L), plum juice (84.66 ± 5.37 mg/L), and fresh orange juice (86.48 ± 8.27 mg/L) dramatically increased the concentration of acetaldehyde in blood, while there was no beverage that could significantly decrease the level of acetaldehyde.

The treatment of honey citron tea significantly decreased the concentration of ethanol in blood of intoxicated mice, and increased the acetaldehyde level in blood significantly, which means that honey citron tea could accelerate the speed of first pass of ethanol metabolism. That is, ethanol could be metabolized rapidly to acetaldehyde, but the acetaldehyde is slowly metabolized to acetic acid. In addition, red bull, fresh orange juice, and plum juice weakly decreased the content of ethanol and apparently increased the acetaldehyde in blood. It was suggested that the second phase of ethanol metabolism was inhibited more potently than the first phase, which led to the accumulation of acetaldehyde in blood. It is well established that acetaldehyde plays a key role in the toxic effects of ethanol [11]. People would get drunk and have hangover symptoms more easily with higher level of acetaldehyde in blood. In addition, the mechanism of a beverage leading to acetaldehyde accumulation was similar to that of a drug therapy for alcohol dependence. Disulfiram, an aldehyde dehydrogenase inhibitor, was approved by the FDA in 1951 as an aversive therapy for treating alcohol dependence. It could block the oxidation of ingested alcohol at the acetaldehyde stage, preventing its rapid and efficient metabolism to acetic acid. As a result, acetaldehyde accumulation caused unpleasant symptoms such as tachycardia, hypotension, diaphoresis, flushing, dyspnea, nausea, and vomiting, acting as a deterrent to alcohol ingestion [15]. Therefore, those beverages which could increase the level of acetaldehyde could be developed to diet supplements to treat alcohol dependency.

### 2.2. Effects of Beverages on Hepatic ADH and ALDH Activities

The ADH and ALDH are two key catalytic enzymes in the alcohol metabolism in liver. As can be seen from Table 2, most of the beverages did not markedly affect the activities of ADH and ALDH in liver. Green tea (14.26% ± 1.24%, *p* < 0.05) and honey chrysanthemum tea (7.78% ± 0.41%, *p* < 0.05) obviously increased the activity of ADH in liver. Iced black tea (21.20% ± 1.79%, *p* < 0.05) and soda water (21.43% ± 3.16%, *p* < 0.05) remarkably boosted the activity of ALDH in liver, while coca cola (−29.98% ± 10.27%, *p* < 0.05), water chestnut juice (−30.90% ± 3.56%, *p* < 0.05), jasmine tea (−41.56% ± 17.17%, *p* < 0.05), and fresh orange juice (−43.71% ± 10.12%, *p* < 0.05) notably inhibited the activity of ALDH in liver.

As above, ethanol was oxidized to acetaldehyde by ADH, mainly in liver; and acetaldehyde was metabolized into non-toxic acetic acid by ALDH. When the ADH activity was boosted, it could accelerate the oxidation of ethanol to acetaldehyde, the ethanol level would decrease and the acetaldehyde level would increase accordingly. When the ALDH activity was inhibited, the speed of acetaldehyde oxidative into acetic acid was slowed, thus the acetaldehyde could be accumulated in the body system, leading to serious hangover symptoms. In brief, the concentrations of ethanol and acetaldehyde in blood were dependent on the relative reaction rate of two major catalyzing enzymes in liver, ADH, and ALDH.

The ADH activity was markedly increased by green tea and honey chrysanthemum tea, which means that they could promote the first pass of alcohol oxidation. In addition, iced black tea and soda water significantly increased the activity of ALDH in liver. Acetaldehyde, the metabolite of ethanol, was transferred to acetic acid by ALDH in many tissues. Agents which could boost the activity of ALDH have the potential to accelerate conversion of acetaldehyde to acetic acid, which has protective health effects. Therefore, green tea, honey chrysanthemum tea, iced black tea, and soda water might provide potential health benefits after excessive alcohol consumption. On the contrary, coca cola, water chestnut juice, jasmine tea, and fresh orange juice evidently inhibited the activity of ALDH, which might contribute to the acetaldehyde accumulation in blood.

**Figure 1:**
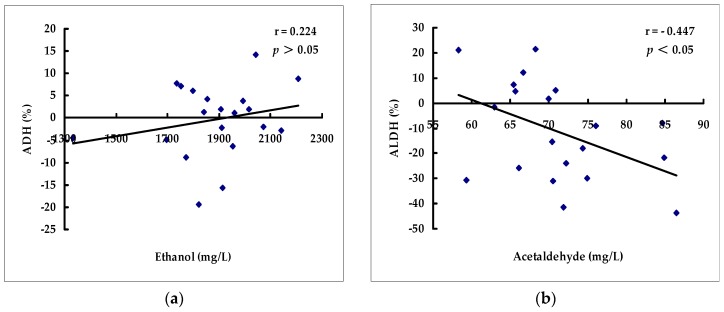
The correlations between the activity of ADH in liver and the content of ethanol in blood (**a**) as well as the activity of ALDH in liver and the content of acetaldehyde in blood (**b**) as influenced by the 20 selected beverages.

In this study, the relationships between the content of ethanol in blood and the activity of ADH in liver as well as acetaldehyde content and ALDH activity as influenced by the 20 selected beverages were obtained and were exhibited in Figure 1. The correlation was analyzed by statistic software SPSS 20.0. As shown in Figure 1b, a moderate negative correlation between acetaldehyde content and ALDH activity was obtained with the correlation coefficient of −0.447 (*p* < 0.05), suggesting that the content of acetaldehyde in blood could be different from the activity of ALDH influenced by beverages. The content of acetaldehyde in blood was decreased when ALDH activity was boosted; on the contrary, the content of acetaldehyde in blood was increased when ALDH activity was inhibited by beverages. Chronic alcohol consumption reduced the activity of ALDH significantly which led to accumulation of acetaldehyde in blood and brain [16]. Acetaldehyde might play an important role in mediating the neuropharmacological and behavioral effects of ethanol.

However, there was no significant correlation between the content of ethanol in blood and the activity of ADH (*r* = 0.224, *p >* 0.05), which indicated that ADH activity in liver might not be responsible for content of blood ethanol after drinking alcohol and these beverages. There are three major enzymes involved in the metabolism of alcohol, that is ADH, catalase (peroxisomes), and P450 2E1 (microsomes) [17]. The ADH metabolized the bulk of ethanol within the liver, and the others also contributed to the production of acetaldehyde from ethanol oxidation. The results suggested that these beverages that influenced the concentrations of ethanol and acetaldehyde in blood were not by means of changing ADH and ALDH activities, but through other ways, such as non-enzymatic ways, which could inhibit the absorption of ethanol in the stomach and intestine or accelerate the excretion of ethanol through breath and urine.

Therefore, beverages such as iced black tea and soda water which could boost the ALDH activity so as to decrease the content of acetaldehyde in blood might possess the capacity of accelerating the metabolism of alcohol and be beneficial in reducing the harm from excessive alcohol consumption. On the contrary, due to the evident inhibition of ALDH activity, the groups of coca cola, honey citron tea, water chestnut juice, jasmine tea, and fresh orange juice showed higher level of acetaldehyde in blood than the control. The results suggested that these beverages might increase the potency of the acetaldehyde toxicity. Red bull and plum juice markedly increased the acetaldehyde in blood might through other mechanism such as inhibition of other enzymatic pathways or non-enzymatic pathways [8,17]. These beverages could disturb the metabolism of ethanol and accumulated toxic acetaldehyde in blood, causing harmful effects on human health.

### 2.3. Effects of Beverages on ALT and AST Levels in Serum

The liver damage was evaluated by measuring the activities of AST and ALT in serum. As showed in Table 3, there was a significant rise of levels of AST and ALT in serum 6 h after alcohol consumption (control group) when compared with the normal group. In the literature [18,19,20], 6 h were usually adopted for evaluation of AST and ALT levels, and some researchers even found that the level of ALT was the highest among 1.5, 3, 6, 12 h [21]. For treatment groups, ban sha herbal infusion, sprite, and fresh orange juice obviously increased the level of AST, while green tea, honey chrysanthemum tea, jasmine tea, and soda water markedly decreased the level of AST in serum. In addition, red bull and fresh orange juice notably increased the level of ALT, while green tea, honey chrysanthemum tea, fructus cannabis herbal infusion, and soda water dramatically lowered the level of ALT in serum.

AST and ALT are enzymes with high concentrations in the cytoplasm. When liver cells were injured, these enzymes leaked into the blood stream and their levels in serum were significantly elevated. Assessment of liver function could be performed by measuring the activities of serum enzymes. ALT mainly presents in liver, whereas AST can be found in liver, skeletal muscle, and cardiac muscle. As a result, ALT is more specific for hepatic damage with respect to AST. The elevated levels of AST and ALT in serum were significantly reduced in the animals groups treated with green tea, honey chrysanthemum tea, and soda water, which suggested that these beverages could provide a protection to alcohol-induced liver damage. Conversely, fresh orange juice caused a marked increase in both serum AST and ALT activities, which indicated that it could aggravate liver injury caused by alcohol. Red bull obviously increased the level of ALT, while ban sha herbal infusion and sprite increased the level of AST. Due to ALT could reflect the liver damage more specific than AST, red bull would do more harm to liver than ban sha herbal infusion and sprite. In addition, jasmine tea decreased the level of AST, and fructus cannabis herbal infusion decreased the level of ALT, suggesting that they might provide some protection from liver injury caused by alcohol.

### 2.4. Effects of Beverages on SOD and MDA Levels in Liver

The effects of the 20 selected beverages on the levels of SOD and MDA in liver were showed in Table 4. In comparison with normal group, there was a significant increase in the level of MDA in control group, suggesting the development of peroxidation in liver tissue. Generally, the majority of these beverages did not obviously affect the levels of MDA and SOD in liver. As can be seen in Table 4, the treatment of soda water, honey chrysanthemum tea, he qi zheng herbal infusion and semen coicis herbal infusion significantly prevented the increase of the MDA level in liver. Jia duo bao herbal infusion and semen coicis herbal infusion decreased the level of hepatic SOD.

Oxidative stress is considered to play a critical role in the pathogenesis of various liver disorders [22]. The formation of acetaldehyde in alcohol metabolism is associated with an increase in reactive oxygen species formation, leading to the development of oxidative stress in the liver. Free radicals derived from oxygen were an important factor to liver injury. The content of malondialdehyde in liver tissues reflex the oxidative stress associated with the ingestion of alcohol, which could be used as an indirect measurement of cellular oxidative injury. Soda water, honey chrysanthemum tea, he qi zheng herbal infusion, and semen coicis herbal infusion significantly decreased the level of MDA in liver, suggesting that these beverages could attenuate alcohol-induced oxidative stress. There was no marked change of SOD in most groups except that jia duo bao herbal infusion and semen coicis herbal infusion lowered the level of SOD. Their antioxidant capacity might be declined by excessive alcohol consumption.

The concentration of ethanol and acetaldehyde in blood as well as the activities of ADH and ALDH in liver are major indicators of the metabolism of alcohol. The reduction in levels of AST and ALT by the beverages is an indication of repair of hepatic tissue damage caused by alcohol, and the levels of MDA and SOD could reflect the extent of peroxidation damage. Over-consumption of alcohol could increase the activities of AST and ALT as well as the content of MDA (Table 3 and Table 4). Three (soda water, honey chrysanthemum tea and green tea) out of the 20 kinds of beverages were recommended to consume after excessive alcohol drinking due to their protection from alcohol-induced injuries. Soda water could significantly decrease the levels of AST, ALT, and MDA, and remarkably increase the activity of ALDH. Honey chrysanthemum tea could significantly decrease the levels of AST, ALT, and MDA and markedly increase the activity of ADH. Green tea could significantly decrease the activities of AST and ALT, increase the activity of ADH. Tea (*Camellia sinensis*) is the most consumed beverage in China. Soda water is an alkalescent soft drink, which is widely used by people for drinking. A study has demonstrated the alkalescent electrolyzed-reduced water has an effect of alcohol detoxification through its antioxidant capacity and has potentiality for relief of ethanol-induced hangover symptoms in Sprague-Dawley rats, such as lowering the levels of AST and ALT in serum, significantly increased the levels of glutathione, glutathione peroxidase, glutathione-S-transferase, Cu/Zn-superoxide dismutase, and catalase in liver [23]. Green tea possessed a strong antioxidant capacity owing to abundant of polyphenols, such as catechin, epicatechin and gallate [24]. Fu *et al.* [25] have evaluated the total phenolic contents and antioxidant capacities of herbal and tea infusions, and the results showed that green tea had high FRAP values (12.722 ± 0.698 g GAE/L), TEAC values (7.853 ± 0.126 g GAE/L) and total phenolic contents (0.660 ± 0.016 g GAE/L). Ingestion of green tea with solid contents 2.0 g/L significantly increased reducing power in plasma [26,27]. Green tea lowered the AST and ALT in this study, which was in accordance with the literature. A cross sectional study in a Japanese population showed that green tea consumption was significantly associated with lower circulating levels of aminotransferases [28].

Two out of the 20 kinds of beverages were not recommended to consume after alcohol drinking. Fresh orange juice significantly increased the content of acetaldehyde in blood as well as the activities of AST and ALT, and remarkably inhibited the activity of ALDH. Red bull could significantly increase the concentration of acetaldehyde in blood and the activity of ALT. It would be better not to drink these beverages simultaneously after alcohol consumption. Studies have confirmed that combining energy drinks (such as red bull) with alcohol could mask the signs of alcohol intoxication, resulting in greater levels of alcohol intake, dehydration, more severe and prolonged hangovers, and alcohol poisoning [29,30].

## 3. Materials and Methods

### 3.1. Chemicals and Reagents

NAD^+^ was purchased from Sigma Chemical Co. (St. Louis, MI, USA). Ethanol, acetaldehyde, Tris, pyrazole, acetic acid, sodium chloride, and chloral hydrate were purchased from Tianjin Chemical Factory (Tianjin, China). Acetaldehyde was redistilled before use. All of the other chemicals and solvents used in this study were of analytical grade. Detection kits were purchased from Jiancheng Institute of Biotechnology (Nanjing, China) and Beyotime Institute of Biotechnology (Shanghai, China).

### 3.2. Beverages

The 20 selected herbal infusions, tea, and carbonated beverages were bought from local markets in Guangzhou, China, which are commercial preparations and in the form of tins containing an aqueous solution. The herbal infusions have been centrifuged to remove particles at 4500 *g* for 5 min, and then stored at 4 °C for using within 1–2 days.

### 3.3. Detection of the Effects of 20 Beveragess on Alcohol Metabolism in Mice

#### 3.3.1. Animal Study for Evaluation of Ethanol and Acetaldehyde Levels in Blood as well as ADH and ALDH Activities in Liver

Seven-week-old male Kunming mice were purchased from Laboratory Animal Center of Sun Yat-Sen University (Guangzhou, China). They were housed in a pathogen-free barrier facility accredited by the Association for Assessment and Accreditation of Laboratory Animal Care and had free access to food and tap water. All procedures involving laboratory animal use were approved by the Institutional Animal Care and Use Committee of Sun Yat-Sen University.

The mice were randomly divided into different groups with five mice in each group, including normal group (untreated group), control group and treatment groups. The mice in treatment groups were received one single dose of 52% ethanol solution (4 g ethanol/kg body weight) and beverage (10 mL/kg) intragastrically, while the control group received the same ethanol solution and corresponding distilled water, and normal group only received corresponding distilled water. Two hours later, the mice were anesthetized by injecting intraperitoneally 10% chloral hydrate (350 mg/kg body weight). Blood and liver tissues were taken from each animal. Liver tissues were frozen immediately for the following biological analyses.

#### 3.3.2. Determination of Concentrations of Ethanol and Acetaldehyde in Blood

Blood samples (0.3 mL) were collected from the eyeball into 8 mL headspace vial which contained 1.2 mL 0.6 mol/L perchloric acid, 0.5 mL 10% trichloroacetic acid and 0.3 mL internal standard (160 mg/L tertiary butanol) for analyzing the concentrations of ethanol and acetaldehyde by using headspace-gas chromatography method.

Quantification of ethanol and acetaldehyde by GC was performed according to the method described by Isse *et al.* [31,32]. The vials containing samples were placed in an electric-heated thermostatic water bath at 70 °C for 30 min, then 500 μL headspace gas was drawn by syringe and injected rapidly to GC column. The injection port temperature was 220 °C, while the flame ionization detector (FID) temperature was 250 °C. The column oven temperature was kept at 40 °C for 5 min, and then programmed to increase from 40 to 240 °C at a rate of 40 °C/min. The flow rate of the carrier gas (nitrogen) was 0.4 mL/min (split ratio 25:1).

#### 3.3.3. Analyses of ADH and ALDH Activities

The liver tissues were weighed and homogenized at a 1:10 ratio (*w*/*v*) at 4 °C in 0.1 mol/L Tris-HCl (pH 7.0), then were centrifuged at 13,000 *g*, 4 °C for 40 min, and the supernatant was obtained for analyzing the activities of ADH and ALDH.

ADH activity was determined by the modified method of Wu *et al.* [33]. In brief, 0.1 mL of 0.3 mol/L ethanol and 2.8 mL 0.0021 mol/L NAD^+^ (prepared by 0.1 mol/L Tirs-HCl buffer, pH 7) were mixed at 25 °C, and then 0.1 mL of the liver supernatant was added to initiate the reaction. The absorbance was immediately measured at 340 nm, and was measured again after 10 min.

ALDH activity was determined by the modified method of Lindahl [34]. Briefly, 0.05 mL of 0.3 mol/L ethanol, 0.1 mL of 10% pyrazole and 2.8 mL 0.0021 mol/L NAD^+^ (prepared by 0.1 mol/L Tirs-HCl buffer, pH 7) were mixed at 30 °C, and then 0.1 mL of the liver supernatant was added to initiate the reaction. The absorbance was immediately measured at 340 nm, and was measured again after the mixture was warmed at 30 °C for 15 min.

The absorbance in the absence of liver supernatant was subtracted as the blank. The hepatic ADH and ALDH activities were determined by monitoring the rate of NADH oxidation in the presence of acetaldehyde in cuvettes. The activity was expressed as a percentage compared to the control.

### 3.4. Detection of the Effects of 20 Beveragess on Acute Alcohol-induced Liver Injury in Mice

#### 3.4.1. Animal Study for Evaluation of ALT and AST Activities in Serum as well as SOD and MDA Levels in Liver

The mice were randomly divided into different groups with five mice in each group, including the normal group (untreated group), control group, and treatment groups. The mice in the control group and treatment groups were administered intragastrically with one single dose of 52% ethanol solution (6 g ethanol/kg body weight), because the most commonly used amount of ethanol to mimic human binge drinking was 5–6 g/kg bodyweight, which corresponds to 0.75 L of whiskey (40% *v*/*v*) in a 75 kg human [35]. Thirty minutes later, treatment groups were received beverage (12 mL/kg), while the control group received the corresponding distilled water. The normal group only received corresponding distilled water. Six hours later, the mice were anesthetized by injecting intraperitoneally 10% chloral hydrate (350 mg/kg body weight) [20,21,36]. Blood and liver tissues were immediately obtained. Blood was placed at room temperature for 1 h, and then centrifuged at 6000 *g* for 10 min to separate serum. The livers were stored at −22 °C for analysis.

#### 3.4.2. Determination of ALT and AST Levels in Serum

The levels of serum ALT and AST were determined by the Jiancheng Bioengineering Institute reagents (Nanjing, China), following the instructions of the manufacturer.

#### 3.4.3. Measurement of SOD and MDA Levels in Liver

Liver tissue samples were taken from the left liver lobe, and then were homogenized in ice-cold 0.9% NaCl solution. The homogenate (10%, *w*/*v*) was centrifuged at 4500 *g* for 10 min and the supernatant was used for biochemical analysis. The levels of MDA and SOD were measured using commercial kits (Jiancheng Institute of Biotechnology, Nanjing, China), in accordance with the manufacturer′s instructions.

### 3.5. Statistical Analysis

All values were presented as means ± SD. Differences between treatment group and the control group were analyzed by independent *t* test using the SPSS 20.0 statistical software. *p <* 0.05 was considered significantly different.

## 4. Conclusions

The effects of 20 selected beverages on alcohol metabolism were evaluated *in vivo*. Generally, effects of the beverages on alcohol metabolism and alcohol-induced liver injury were very different. Soda water, green tea, and honey chrysanthemum tea are recommended to consume accompanied with alcohol drinking due to their capacities of accelerating ethanol metabolism and preventing liver injuries caused by alcohol. They might be potential dietary supplement for the alleviation of harmful effects from alcohol consumption. On the contrary, some beverages are not advised to drink accompanied with alcohol consumption, such as fresh orange juice and red bull, due to their adverse effects on ethanol induced liver injury. This study has supplied new information on the effects of the beverages on alcohol metabolism for nutritionists and the general public, and further studies on the precise compounds and mechanisms responsible for the action is needed.

## Figures and Tables

**Table 1:** Effects of the 20 selected beverages on ethanol and acetaldehyde level in blood.

Group	Ethanol Level (mg/L)	Acetaldehyde Level (mg/L)
Normal group	147.11 ± 14.87	25.35 ± 4.07
Control group	1901.17 ± 296.83 **	69.17 ± 5.33 **
Ban sha herbal infusion	1821.83 ± 182.49	66.09 ± 15.36
Coca cola	1909.29 ± 231.45	74.99 ± 4.93
Fresh orange juice	1696.53 ± 170.31	86.48 ± 8.27 *
Fructus cannabis herbal infusion	2140.80 ± 467.26	74.35 ± 4.96
Green tea	1862.48 ± 401.14	65.39 ± 7.83
He qi zheng herbal infusion	2208.69 ± 406.42	72.22 ± 3.81
Honey chrysanthemum tea	1734.96 ± 272.68	66.70 ± 4.78
Honey citron tea	1332.72± 249.50 *	84.88 ± 16.75 *
Honey jasmine tea	1853.08 ± 384.00	65.62 ± 5.23
Iced black tea	1797.09 ± 301.41	58.26 ± 4.01
Jasmine tea	1960.09 ± 220.74	71.87 ± 7.57
Jia duo bao herbal infusion	2073.84 ± 292.95	70.82 ± 8.80
Plum juice	1752.74 ± 214.60	84.66 ± 5.37 *
Red bull	1907.48 ± 312.00	76.12 ± 2.60 *
Rock candy pear juice	1842.41 ± 148.05	62.93 ± 3.63
Soda water	1951.82 ± 237.80	68.22 ± 4.21
Semen coicis herbal infusion	1773.14 ± 169.46	59.32 ± 6.51
Sprite	1737.21 ± 384.33	70.45 ± 4.73
Water chestnut juice	2014.97 ± 233.86	70.51 ± 1.84
Wang lao ji herbal infusion	1992.95 ± 182.08	69.94 ± 3.86

*: Difference between the sample and the control was statistically significant (*p* < 0.05); **: Difference between the control and normal group was statistically significant (*p* < 0.05).

**Table 2:** Effects of the 20 selected beverages on ADH and ALDH activity in liver.

Group	ADH (%)	ALDH (%)
Ban sha herbal infusion	−19.47 ± 5.40	−25.81 ± 5.41
Coca cola	−2.09 ± 0.21	−29.98 ± 10.27 *
Fresh orange juice	−4.80 ± 0.26	−43.71 ± 10.12 *
Fructus cannabis herbal infusion	−2.82 ± 0.18	−18.08 ± 5.33
Green tea	14.26 ± 1.24 *	7.42 ± 2.32
He qi zheng herbal infusion	8.86 ± 0.55	−23.94 ± 4.95
Honey chrysanthemum tea	7.78 ± 0.41 *	12.26 ± 3.18
Honey citron tea	−4.37 ± 0.22	−21.68 ± 6.62
Honey jasmine tea	4.20 ± 0.60	4.69 ± 0.70
Iced black tea	6.11 ± 0.08	21.20 ± 1.79 *
Jasmine tea	1.14 ± 0.17	−41.56 ± 17.17 *
Jia duo bao herbal infusion	−2.02 ± 0.17	5.08 ± 1.53
Plum juice	1.34 ± 0.22	−1.56 ± 0.82
Red bull	1.98 ± 0.24	−8.89 ± 1.54
Rock candy pear juice	7.20 ± 0.48	−7.85 ± 2.48
Soda water	−6.39 ± 1.48	21.43 ± 3.16 *
Semen coicis herbal infusion	−8.85 ± 0.93	−30.64 ± 0.79
Sprite	−15.64 ± 3.81	−15.33± 3.14
Water chestnut juice	2.05 ± 0.11	−30.90 ± 3.56 *
Wang lao ji herbal infusion	3.78 ± 0.55	1.64 ± 0.20

*: Difference between the sample and the control was statistically significant (*p* < 0.05).

**Table 3:** Effects of the 20 selected beverages on serum AST and ALT activities in serum.

Group	AST	ALT
Normal group	40.11 ± 9.04	20.52 ± 7.14
Control group	55.28 ± 11.23 **	35.88 ± 11.04 **
Ban sha herbal infusion	101.68 ± 35.2 *	34.10 ± 7.09
Coca cola	55.20 ± 13.91	35.36 ± 11.27
Fresh orange juice	77.97 ± 13.24 *	49.01 ± 6.74 *
Fructus cannabis herbal infusion	51.59 ± 14.06	19.91 ± 7.06 *
Green tea	20.91 ± 9.95 *	20.81 ± 1.70 *
He qi zheng herbal infusion	49.54 ± 32.66	32.18 ± 8.99
Honey chrysanthemum tea	10.53 ± 2.43 *	28.09 ± 1.24 *
Honey citron tea	41.42 ± 10.40	38.45 ± 5.89
Honey jasmine tea	59.65 ± 15.89	32.94 ± 12.46
Iced black tea	46.44 ± 11.50	33.14 ± 9.91
Jasmine tea	32.03 ± 9.04 *	38.45 ± 11.40
Jia duo bao herbal infusion	42.03 ± 17.06	25.44 ± 6.33
Plum juice	43.00 ± 14.90	36.24 ± 14.39
Red bull	57.03 ± 24.72	63.55 ± 17.87 *
Rock candy pear juice	31.81 ± 11.04	28.80 ± 2.18
Soda water	16.31 ± 15.88 *	25.11 ± 1.76 *
Semen coicis herbal infusion	55.27 ± 11.49	28.06 ± 8.04
Sprite	78.15 ± 4.41 *	38.15 ± 15.01
Water chestnut juice	55.38 ± 11.41	31.18 ± 6.21
Wang lao ji herbal infusion	69.43 ± 15.75	40.27 ± 9.81

*: Difference between the sample and blank was statistically significant (*p* < 0.05). **: Difference between the control and normal group was statistically significant (*p* < 0.05).

**Table 4:** Effects of the 20 selected beverages on hepatic MDA and SOD levels in liver.

Group	MDA (nmol/mg protein)	SOD (U/mL)
Normal group	0.75 ± 0.07	60.51 ± 3.66
Control group	0.86 ± 0.16 **	57.83 ± 7.62
Ban sha herbal infusion	0.96 ± 0.30	56.78 ± 2.75
Coca cola	0.84 ± 0.18	68.34 ± 10.00
Fresh orange juice	0.83 ± 0.23	55.61 ± 3.95
Fructus cannabis herbal infusion	0.74 ± 0.21	53.53 ± 4.45
Green tea	0.87 ± 0.15	60.52 ± 5.40
He qi zheng herbal infusion	0.55 ± 0.10 *	51.57 ± 4.19
Honey chrysanthemum tea	0.68 ± 0.06 *	61.98 ± 5.34
Honey citron tea	0.76 ± 0.08	55.63 ± 8.50
Honey jasmine tea	0.75 ± 0.10	59.94 ± 2.80
Iced black tea	0.98 ± 0.29	52.33 ± 6.02
Jasmine tea	0.89 ± 0.13	59.49 ± 4.62
Jia duo bao herbal infusion	0.79 ± 0.32	52.90 ± 3.59 *
Plum juice	0.75 ± 0.08	58.82 ± 7.43
Red bull	0.78 ± 0.41	57.03 ± 3.20
Rock candy pear juice	0.66 ± 0.15	55.67 ± 4.53
Soda water	0.54 ± 0.10 *	58.52 ± 6.29
Semen coicis herbal infusion	0.56 ± 0.19 *	51.34 ± 2.87 *
Sprite	0.76 ± 0.14	57.61 ± 1.77
Water chestnut juice	1.15 ± 0.27	61.49 ± 7.51
Wang lao ji herbal infusion	0.77 ± 0.24	58.19 ± 9.43

*: Difference between the sample and blank was statistically significant (*p* < 0.05). **: Difference between the control and normal group was statistically significant (*p* < 0.05).
